# Bioremediation of lunar regolith simulant through mycorrhizal fungi and plant symbioses enables chickpea to seed

**DOI:** 10.1038/s41598-026-35759-0

**Published:** 2026-03-05

**Authors:** Jessica Atkin, Elizabeth Pierson, Terry Gentry, Sara Oliveira Santos

**Affiliations:** 1 Department of Soil and Crop Sciences, Texas A&M University, College Station, TX 77843 USA; 2 Department of Horticultural Sciences, Texas A&M University, College Station, TX 77843 USA; 3 Center for Fluid Mechanics, School of Engineering, Brown University, Providence, RI 02912 USA; 4 Institute for Geophysics, Jackson School of Geosciences, University of Texas at Austin, Austin, TX 78712 USA

**Keywords:** Space crops, Horticulture, In-situ resource utilization, Lunar regolith simulant, Ecology, Ecology, Environmental sciences, Microbiology, Plant sciences

## Abstract

Food sustainability is a significant challenge for long-term space travel. Plants can provide fresh nutrition, reducing reliance on packaged foods. Using Lunar regolith simulant (LRS), we tested a methodology to create a productive growth medium for horticultural crops on the Moon. We leveraged chickpea (*Cicer arietinum*), Arbuscular Mycorrhizal Fungi (AMF), and Vermicompost (VC) to enhance plant stress tolerance, sequester contaminants, and improve substrate structure. Chickpeas were cultivated in LRS/VC mixtures, with or without AMF, under climate-controlled conditions. Plants seeded successfully in mixtures containing up to 75% LRS when inoculated with AMF. While the number of seeds declined with increasing LRS concentration, seed size remained stable. Higher LRS concentrations induced stress; however, plants grown in 100% LRS inoculated with AMF demonstrated an average extension of two weeks in survival compared to non-inoculated plants. AMF colonized roots across all mixtures, including 100% LRS, demonstrating the ability to establish symbioses under extreme conditions. We also observed improvement in the structural properties of LRS by forming aggregates capable of withstanding extreme conditions, potentially mitigating particle-related hazards. These results provide a baseline for chickpea establishment and yield in amended LRS while demonstrating biological improvements in regolith properties.

## Introduction

To return humans to the Moon and establish a Lunar presence, we must maximize in situ resources and use Lunar regolith (LR) and regenerative processes to provide a sustainable support substrate for horticultural crops^1–5^. The use of LR as the sole growing medium presents challenges due to toxic elements, exposure to cosmic and solar radiation, lack of organic materials, absence of rhizosphere microorganisms, and poor structural properties^1,6,7^. However, LR contains essential nutrients for plant growth, including Phosphorus (P), Potassium (K), Calcium (Ca), Magnesium (Mg), and Iron (Fe)^1^. In addition, Silicon (Si) and Titanium (Ti), abundant in LR, can benefit plants by enhancing structural integrity and promoting stress tolerance. A key nutrient, nitrogen (N) is scarce and will require supplementation. Although Lunar regolith contains essential nutrients, most are bound in mineral forms that are poorly bioavailable to plants. The introduction of Earth-derived rhizosphere microorganisms can enhance nutrient solubilization through biological weathering, organic acid production, and enzyme activity, thus improving the suitability of regolith as a plant growth medium.

Experiments using LR samples returned during the Apollo missions showed that seeds can germinate successfully after short-term exposure to irradiated Lunar samples^8–10^. Research using *Arabidopsis thaliana* showed that although plants can germinate in LR, they exhibit slower development and severe stress morphologies^11^. Long-term exposure to metals found in Lunar regolith (LR), such as Iron (Fe), Aluminum (Al), Zinc (Zn), and Copper (Cu), can contribute to plant toxicity, severely altering physiological processes and causing oxidative damage. Although Ca and Mg are essential for plant growth, their concentrations must be carefully managed, as excessive levels can be damaging to plants. Furthermore, Lunar regolith has poor physical and structural properties. Water permeability of Lunar highland regolith is one to two orders of magnitude lower than that of fine silica sands due to the irregular and angular shape and hydrophobicity of LR particles^12^. These properties, in addition to particle size (median particle size between 40–$130\,\upmu \text {m}$) and poor aggregate development pose challenges to water retention, nutrient availability, and gas exchange^1^. Additionally, Lunar regolith lacks a microbiome, crucial for nutrient transformation and plant uptake, preventing efficient nutrient conversion and plant availability. These combined factors highlight some of the limitations of cultivating plants using a Lunar-based substrate.

**Fig. 1:**
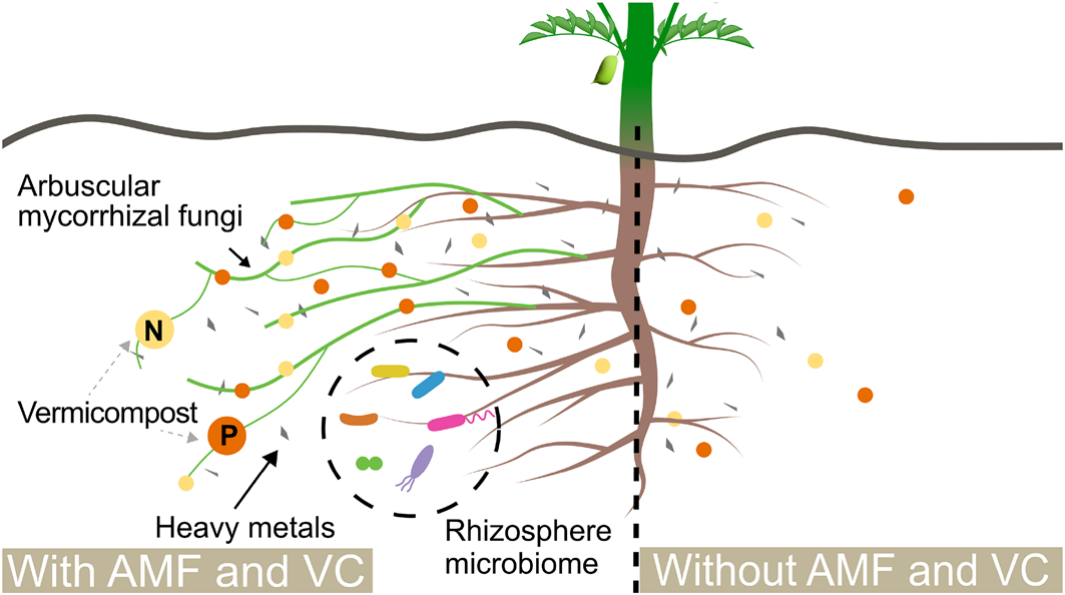
Interactions between chickpea (CP), arbuscular mycorrhizae (AMF), and vermicompost (VC) in the rhizosphere. Chickpea has a taproot reaching up to 30 cm with lateral root branching. The root system with AMF (left) accesses a larger volume of the planting medium, reaching otherwise inaccessible nutrients and water while sequestering heavy metals (HMs) from uptake. The root system without AMF (right) has less surface area, shorter roots, and fewer protective mechanisms against HMs. VC introduces rhizosphere microorganisms and provides nutrients necessary for plant development.

To use LR as a growth medium, we must first initiate a transformation of the LR matrix to enhance its structure and mitigate element toxicity. We can incorporate stable organic amendments to improve the structural properties of regolith and introduce a microbiome. Studies using mixtures of compost and Lunar regolith simulants (LRS) showed that amendments can improve Martian and Lunar regoliths as a plant growth medium by increasing nutrient availability and improving hydraulic properties^13–15^. Due to its physical properties, LRS has proven to be a challenging growth medium, even with compost mixtures. To succeed in using LR as a growth medium, we must change its chemical and physical properties to facilitate the establishment of a microbiome. Here, we focus on overcoming these challenges by creating a fertile support matrix to sustain plant and microbial life using arbuscular mycorrhizal fungi (AMF) and vermicompost (VC), the benefits of which are summarized in Fig. 1.

AMF can remediate heavy metal-contaminated soils through phytoremediation and protect the plant through multiple mechanisms^16^. Exudates from extraradical mycelium are used to sequester heavy metals in the rhizosphere, reducing their bioavailability and preventing them from uptake by the host plant. Together, extracellular polymeric substances on the surface of hyphae work together with other compounds to prevent the entry of heavy metals into the root tips^17,18^. A secondary mechanism allows AMF to accumulate heavy metals in its fungal biomass and the roots of the host plant^19^. This enables them to stimulate plant resistance, reduce the negative impact of heavy metal toxicity, and promote plant growth under conditions of metal stress^20^.

AMF play vital roles in regulating rhizosphere productivity by improving nutrient cycling capabilities and promoting soil aggregation, thereby influencing particle organization, structure, and stabilization.^21–23^. AMF act at different levels to enhance soil stability. AMF hyphae can entangle particles to form aggregates, as well as cause morphological changes in the roots to create compressive stresses that lead to soil compression and reorientation of particles^24–26^. AMF can alter plant carbon metabolism, leading to rhizodeposition, stimulating aggregate formation^27^. In addition, AMF produce glomalin, a glycoprotein that acts as a binding agent, improving aggregate stability and reducing soil erosion^28,29^.

*Vermicomposting* is a bio-oxidative process resulting from the synergistic action of red wiggler earthworms (*Eisenia fetida*) and their gut microbiota to decompose biowaste. The resulting biostimulant is rich in essential plant nutrients and minerals and has a diverse microbiome. Earthworms produce water-soluble nutrients and humus while improving soil aggregate formation, lowering bulk density, modifying soil structure, and improving water retention^30^. Vermicomposting is an effective way to recycle clothing, hygiene items, and food waste found in the NASA logistics reduction and repurposing project to produce a sustainable microbe-rich fertilizer^31^. Although we do not directly use earthworms in this research, we use a by-product of their activity, specifically nutrient-rich vermicompost, to enhance plant growth, LRS structure, and promote healthy rhizosphere.

We use chickpea as our model plant due to its nutritional content, its ability to host symbiosis with AMF^32,33^, and its tolerance to stress. Chickpea is a legume crop high in protein, carbohydrates, iron, phosphorus, calcium, vitamin B, and other nutrients and does not require large water or nitrogen inputs. Chickpea is used globally as a nutritious protein substitute for meat, and has been used in studies evaluating the remediation of radioactive and metal-contaminated soils^34–36^. We hypothesize that the symbiosis created through AMF, vermicompost, and chickpea will provide a sustainable support substrate for Lunar crop production (Fig. 1). We test this hypothesis through experiments growing chickpea plants in varying concentrations of LRS and vermicompost (LRS/VC), with or without AMF. By maximizing interactions between chickpea, AMF, and vermicompost, we aim to create a sustainable, low-input system for safe Lunar crop production. Additionally, the insights gained could be implemented to improve soil health and plant resilience in challenging Earth soils, emphasizing the relevance of this research for both sustainable Moon and Earth based agriculture.

## Results and discussion

In this study, we cultivated chickpea in mixtures of vermicompost and LRS up to 100% LRS, to determine whether the interaction between chickpea, AMF and vermicompost creates a fertile LRS matrix capable of supporting the growth of chickpea to maturity. Treatments included the following mixtures of LRS and vermicompost (VC): 25%LRS / 75% VC (LRS25), 50%LRS / 50% VC (LRS50), 75%LRS / 25% VC (LRS75) and 100% LRS (LRS100) with or without AMF inoculation. Plant growth, development, and health in LRS was compared to plants grown in the potting mix control, with or without AMF.

Plant establishment measurements^37^ were taken on day 28, 21 days after 50% emergence in the potting mix controls. Establishment was 100% in the controls and all LRS treatments for both AMF treated and untreated seeds, indicating that contact with LRS did not interfere with early seedling development stages. Between day 28 and 56, AMF treated and untreated plants in 50%, 75%, and 100% LRS developed signs of stress including stunted growth, loss of leaf area, and reduction or lack of shoot branching. Additionally, leaf yellowing was observed to be higher for plants grown in higher concentrations of LRS. These responses are likely linked to nutrient limitations inherent to LRS, including lack of nitrogen and restricted solubility of phosphorus. Such deficiencies would impair chlorophyll synthesis, reduce leaf expansion, and limit shoot branching, consistent with the symptoms observed. These findings are consistent with previous experiments using anorthosite and Lunar regolith samples returned during the Apollo missions^11,38,39^.

By day 56, there was a clear difference between AMF inoculated and untreated plants in visual health, suggesting successful AMF colonization. The difference was most noticeable between AMF treated and untreated plants grown in 100% LRS, with AMF treated plants being greener and more turgid than untreated plants. All plants grown in 100% LRS died before the end of the experiment, although AMF treatment extended the life of plants. Non-AMF plants began to senesce by day 61, whereas AMF-treated plants did not begin to senesce until day 75, demonstrating that AMF inoculation extended the growth of plants in 100% LRS by two weeks.

**Fig. 2:**
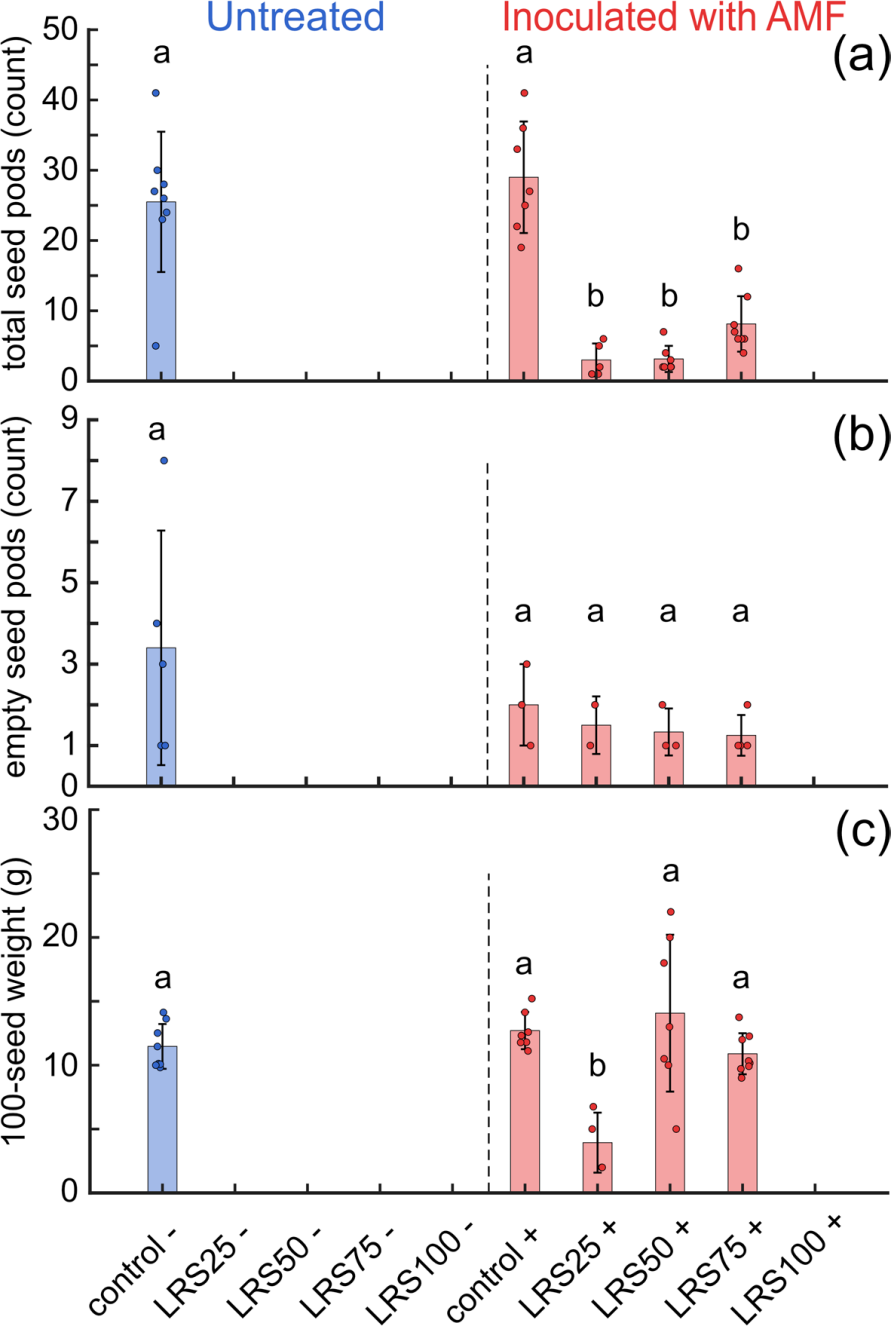
Chickpea total seed count and weight in potting mix controls, LRS/VC mixtures, and 100% LRS with or without (+/-) AMF inoculation. (**a**) Total seed pods per plant. Seed production in both controls was comparable (p-value = 0.7357), whereas yields in LRS25, LRS50, and LRS75 were significantly lower (*p* <0.001). (**b**) Empty seed pods per plant. Empty pod numbers did not differ significantly among treatments with seed set. (**c**) Standardized seed mass (100-seed weight). Significant differences were found only for LRS25 (p-value = 0.0012), while control, LRS50, LRS75, and LRS100 did not differ, showing that although increasing LRS concentrations reduced seed number, seeds that developed were comparable in weight to those in control plants. Bars show mean ± standard error ($n = 8$). Different letters denote significant differences (ANOVA, $p < 0.05$).

Flowering and seed set occurred exclusively in the controls and in plants inoculated with AMF grown in 25%, 50%, and 75% LRS (Fig. 2A–C, Supplementary Fig. 1), demonstrating that AMF inoculation enabled seed production in LRS/VC mixtures. For these treatments, the first flowers were observed on days 70–77. Compared to AMF treated control plants, AMF treated plants grown in LRS mixtures were delayed in seed production and time to full maturity (100 versus 120 days, respectively).

Measuring seed production and weight informs yield and quality (Fig. 2). For long-term space agriculture, plants must not only produce seeds, but produce seeds of sufficient size and viability to support multi-generation cropping. Outside of the potting mix controls, only AMF-inoculated LRS/VC mixtures produced seed. Seed production in the controls with and without AMF was comparable. In contrast, seed yield in LRS25, LRS50, and LRS75 was significantly lower (*p* $<0.001$), respectively), indicating that higher LRS limits reproduction. Empty seed pod levels were not significantly different among treatments. This suggests that once seed set was initiated, pod filling occurred at similar rates. The main limitation was the initiation of seed set rather than the filling process itself.

Standardized 100 seed weight differed only for LRS25 (p-value = 0.0012). Seeds from the control, LRS50 and LRS75 were not significantly different. This means that while higher LRS mixtures reduced the total number of seeds, the seeds that did form were similar in weight to those from the controls. However, future studies must test the metal content of harvested seeds to determine if they are safe for consumption. These results suggest that early stressors such as nutrient limits, poor substrate structure, and water stress restricted reproductive development. AMF colonization may have supported nutrient uptake during later stages, allowing normal seed filling and stabilizing seed mass across treatments.

**Fig. 3:**
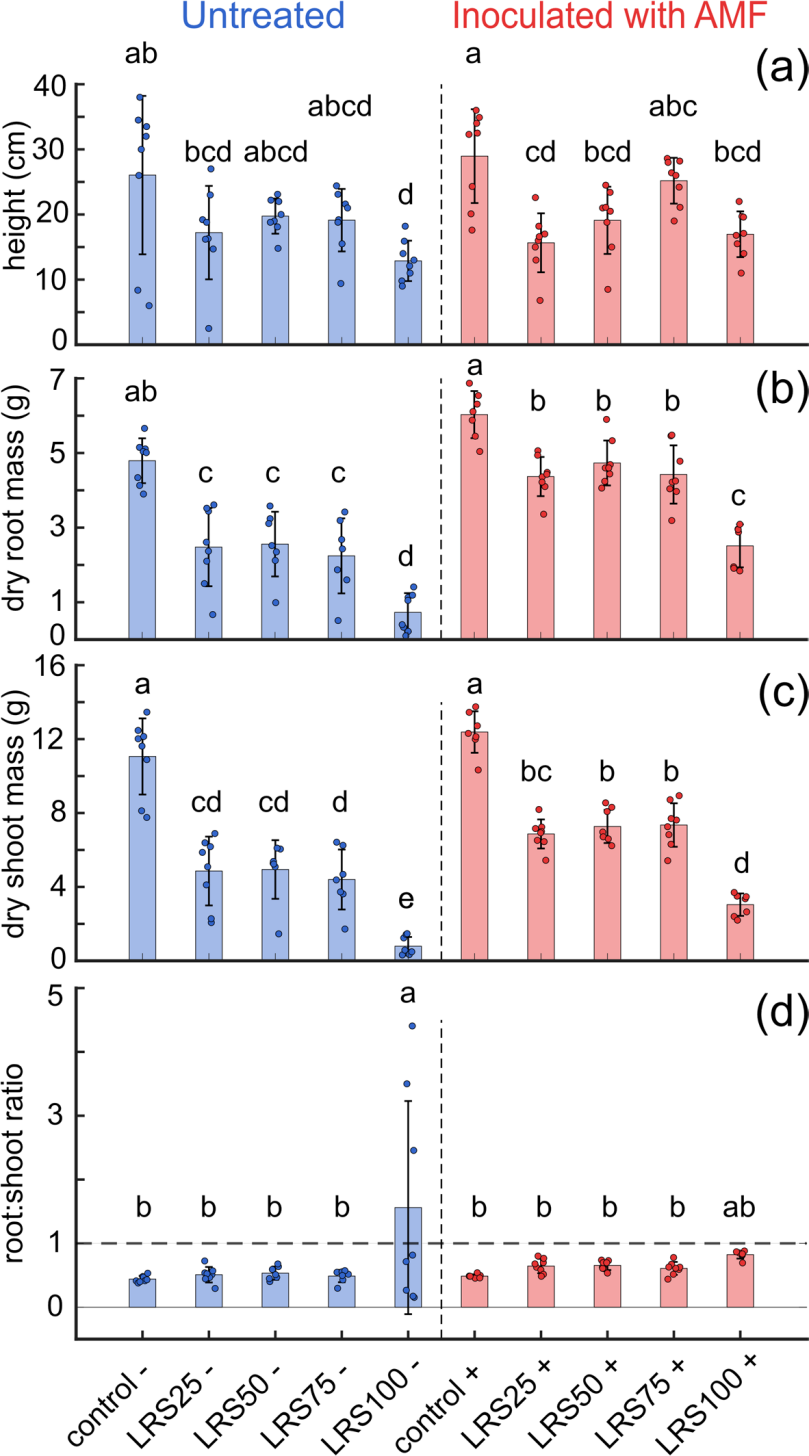
Quantification of plant metrics. (**A**) Plant height. Control plants with or without AMF inoculation ($+/-$, respectively) were taller than most LRS treatments, with height significantly reduced in LRS75 and LRS100, without AMF. (**B**) Root biomass was greatest in controls, while LRS treatments, especially LRS100 without AMF, showed reduced root growth. (**C**) Shoot biomass followed a similar trend, with controls producing the greatest shoot mass and LRS100 without AMF the lowest. (**D**) Ratios clustered near 1 across most treatments, except for LRS100 without AMF, which showed a significantly elevated root:shoot allocation. Bars show mean ± standard error (n = 8). Different letters indicate significant differences (ANOVA, $p < 0.05$).

Plant growth was strongly influenced by substrate composition (Fig. 3a). Control plants had greater height, root mass, and shoot mass compared to all treatments, and these differences were significant for treatments in both uninoculated and AMF-inoculated substrates (Fig. 3). AMF-inoculated substrates had significantly greater dry root mass compared to uninoculated plants, and LRS50+ and LRS75+ showed significantly higher dry shoot mass compared to the uninoculated mixtures (Fig. 3b,c). These patterns suggest that AMF inoculation improved growth. By contrast, growth responses in LRS25+ were weaker, which may be due to excess nutrient availability leading to over-nutrition, as well as the high water-holding capacity of vermicompost creating suboptimal conditions.

Root to shoot ratios remained close to 1 across most treatments, showing proportional allocation between aboveground and belowground tissues (Fig. 3d). Maintaining balanced allocation is important because it supports efficient resource use and sustained growth. An exception occurred in LRS100 without AMF, where the ratio was significantly elevated, reflecting a stress response that favored root growth under substrate limitations. This suggests that while LRS constrained growth, plants generally maintained balanced allocation until stress became severe enough to shift toward root investment. These constraints on vegetative growth carry forward into reproduction, where reduced biomass likely contributed to lower seed yield under higher LRS levels. At harvest, assessment of AMF colonization confirmed that all AMF inoculated plants had successful mycorrhization, including plants grown in 100% LRS, while no colonization was observed in uninoculated treatments. These results demonstrate that AMF mycorrhization occurs in LRS.

Soil transformation metrics, such as pH, are valuable for evaluating LRS as a plant growth medium. Soil pH determines the solubility and bioavailability of essential nutrients as well as the mobility of potentially toxic metals. Most plants thrive in soil within the slightly acidic to neutral range (6.0–7.5) due to the bioavailability of macronutrients in that pH range. However, in Earth soils, metals found in high concentrations in LR, such as iron (Fe), aluminum (Al), zinc (Zn), and copper (Cu), become more available to plants under slightly acidic conditions (pH of 6.0–6.5), and the solubility of these metals increases sharply under more acidic or reducing conditions^40^. For example, Al is considered toxic to roots at a pH below 5.5^41^. It is unclear how LR affects the bioavailability of these metals; however, given the high levels of the metals in LR, acidity may drive levels to a point where they are considered a toxicant^42^. At the start of the study, the pH of 100% LRS was strongly alkaline (pH 9.9, Table 1). In contrast, the pH of the LRS25, LRS50, and LRS75 mixtures was slightly acidic (5.9–6.4), demonstrating the pH-reducing effect of vermicompost on the LRS. After harvest, the AMF-uninoculated LRS/VC mixtures shifted toward more neutral values (6.5–7.3), while AMF-inoculated mixtures experienced less of a pH increase and seemed to stabilize around a narrower, slightly acidic pH range (6.2–6.6). Thus, inoculation with AMF kept the LRS mixtures within the pH window where macro nutrient availability is likely to be high, but contaminating metals are likely to be soluble and bioavailable to the plants and the AMF. It remains unclear the extent to which these metals were incorporated into plant tissues or sequestered by AMF, a distinction that requires further testing.

**Table 1:** The pH values of potting mix, and lunar regolith simulant mixtures at the start of the study ($t_0$) and after harvest without (−) inoculation with ($+$) inoculation of AMF.

Treatment	pH @ $t_0$	pH @ harvest (−)	pH @ harvest (+)
PM	6.1	7.8	7.3
LRS25	5.9	6.5	6.2
LRS50	6.0	6.7	6.4
LRS75	6.4	7.3	6.6
LRS100	9.9	8.8	8.7

**Fig. 4:**
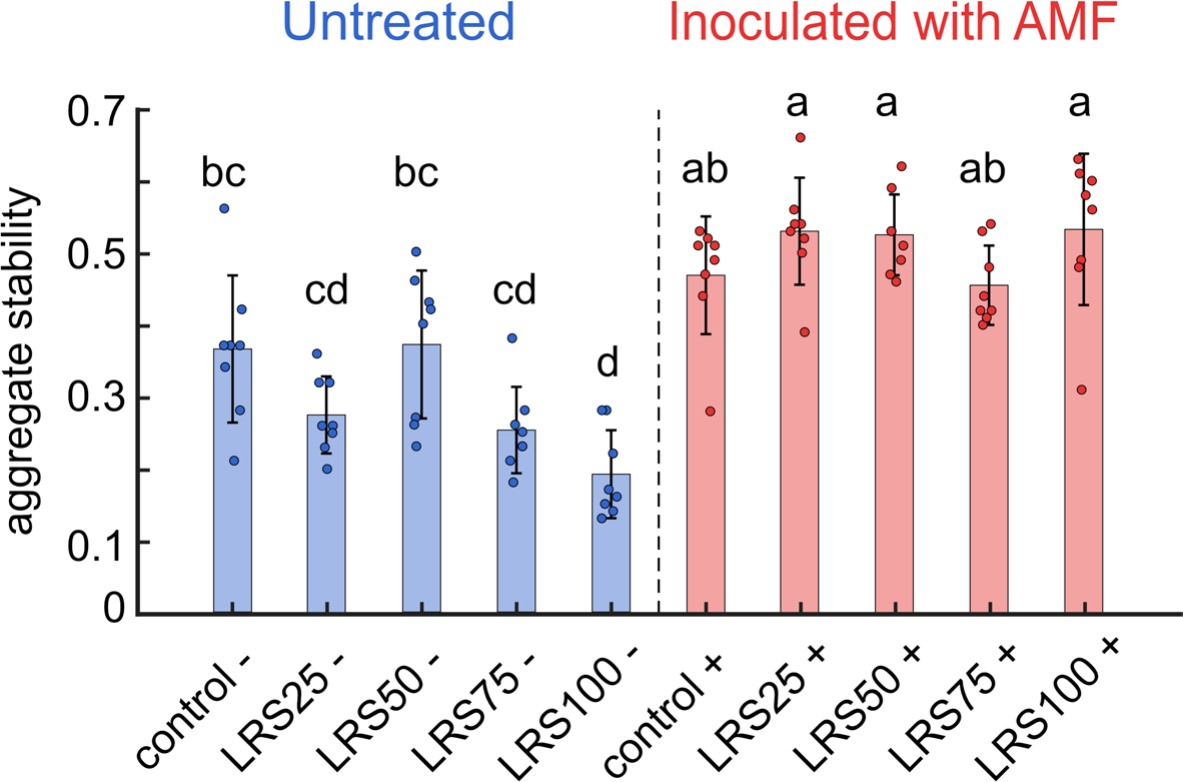
Aggregate stability of chickpea grown in the potting mix control, LRS/VC mixtures, or 100% LRS with or without AMF inoculation measured using SLAKES. AMF inoculation significantly enhanced aggregate stability across all substrates, while uninoculated treatments, particularly LRS100, showed reduced stability. Bars show mean ± standard error with individual replicates overlaid. Letters indicate groups that are not significantly different (ANOVA, $p < 0.05$).

One of the major limitations of Lunar regolith is its structure, which results in poor water retention, limited nutrient mobility, and structural instability. AMF are known on Earth to improve soil structure through hyphal networks and exudate production, processes that directly contribute to aggregate formation. Aggregate stability, measured via the SLAKES test, was significantly greater across all treatments with AMF-inoculated chickpeas (Fig. 4)^43,44^.These results demonstrate AMF-plant symbiosis not only forms successfully in LRS but also improves its structural integrity, addressing a key barrier to developing regolith into a functional growth medium within a single plant generation.

## Conclusion

Using Earth-based soil regeneration techniques, we report the first instance of growing chickpea to seed in LRS. Our results demonstrate that terrestrial soil regeneration strategies, particularly inoculation with AMF, can significantly enhance plant health and facilitate the conditioning of Lunar regolith for agriculture. Somewhat surprisingly, we confirmed that mycorrhization occurred in all AMF-inoculated LRS treatments, including 100% LRS. Additionally, we achieved flowering in the AMF-inoculated mixtures with 25/75, 50/50, and 75/25 LRS/VC composition. While seed count decreased with higher concentrations of LRS, individual seed weight was not affected. Although all the AMF-inoculated plants grown in 100% LRS died before flowering, AMF inoculation extended the life of plants grown by 14 days compared to uninoculated plants grown in 100 LRS. Although these are promising results, all plants showed signs of stress including chlorophyll deficiency, stunting, reduced leaf area, and limited shoot branching. To alleviate the stress, we suggest the need to employ a larger number of generations planted in the conditioned substrate to further improve the LRS for crop growth.

We observed that AMF-inoculated treatments exhibited significantly higher aggregate stability compared to untreated samples reinforcing the role of biological amendments in improving soil structure. These findings provide valuable insights into adaptive responses, emphasizing the potential of AMF to enhance plant resilience and improve the structural integrity of LR. We present a step toward sustainable agriculture on the Moon, addressing the fundamental challenges of using Lunar regolith as a plant growth medium. This investigation advances our understanding of plant-microbe interactions in extraterrestrial environments, highlighting the potential for in situ resource utilization to support future Lunar habitats. Significant challenges remain, including optimizing regolith conditioning processes, mitigating plant stress responses, and exploring long-term soil-microbe-plant interactions under Lunar conditions. Future studies can build on this foundation by enhancing regolith fertility and resilience, exploring diverse crop systems to support sustainable food production for Lunar missions, and incorporating community-level microbiological analyses of rhizosphere and bulk soil species. Our approach recasts the challenges of utilizing Lunar regolith into opportunities for creating a sustainable Lunar habitat.

## Methods

We assessed the impact of regolith, VC, and AMF on chickpea growth in LRS. We used four different mixes of VC and LRS, with and without AMF using a randomized block design (Supplementary Fig. 2). VC (Black Diamond Vermicompost, Paso Robles, CA)  and LRS were mixed by weight ($\rho _{VC} = 0.39 \, \mathrm{g/cm}^3$, $\rho _{LRS} = 1.3 \, \mathrm{g/cm}^3$ ) in the following compositions: LRS25 (25%LRS/ 75%/ VC, n = 8); LRS50 (50%/ LRS 50% VC, n = 8); LRS75 (75% LRS/ 25% VC, n = 8); and LRS100, (100% LRS, n = 8). Half of the compositions (n = 16) were inoculated with Arbuscular Mycorrhizal Fungi (Mycoapply, Grants Pass, OR) containing *Rhizophagus intraradices*, *Funneliformis mosseae*, *Claroideoglomus claroideum* and *Claroideoglomus etunicatum*. Two controls were included using potting mix (PM) (n = 16) (Sungro Professional Potting Mix), one group AMF-inoculated and one not inoculated. Two methods of watering were tested. Top watering of 100% LRS resulted in substrate crusting that impeded infiltration and persisted. To avoid this problem, we adopted bottom watering using cotton wicks that delivered moisture to the substrate by capillary action. We recommend wick-based bottom watering for future experiments with LRS to minimize crust formation and ensure consistent hydration.

Lunar regolith simulant (LRS) was used due to the limited availability of Lunar Regolith obtained from the Apollo missions. LRS is created using geological materials found on Earth to duplicate the physical, mineralogical, and chemical compositions of Lunar regoliths. These simulants approximate the composition of specific Lunar landing sites to include plagioclase, pyroxene, olivine, ilmenite, chromite, and glass from meteor impacts^45^. Here, we use a high-fidelity Lunar regolith simulant, LHS-1 (ExolithLabs, Oviedo, FL), which mimics the Lunar highlands^46^. This LRS replicates the mineralogy, geochemistry, and particle size of LR^47–49^. The chemical composition of this substrate is included as Supplementary Table 1.

The Desi chickpea variety Myles (Territorial Seed Company, Cottage Grove, OR, USA) was chosen for its compact size and tolerance to stress^50^. Plants were grown to full maturity in 35 cm tall containers (total volume of 983 cm³, Deepot Tree Pots) to allow for root expansion over their growth period. Rockwool was affixed in the lower opening of the containers to prevent the loss of small particles. All seeds were planted on an LRS matrix on day 0. Plants were watered immediately after sowing, again on day two, and then every other day using a bottom-wick irrigation system. The wicking setup consisted of 6.35 mm cotton cords connected to water reservoirs, allowing passive and consistent moisture delivery. The control group was watered (using the same wick method) with a nutrient solution containing 75 mg L$^{-1}$ nitrogen and other essential nutrients, prepared using an all-purpose water-soluble fertilizer (12N–1.75P–13.3K; Jack’s Nutrients FeED 12–4–16 RO; JR Peters, Inc., Allentown, PA) dissolved in deionized water, with an additional 10 mg L$^{-1}$ sulfur supplied as magnesium sulfate (MgSO$_4 \cdot$ 7H$_2$O). The study was conducted in a climate-controlled growth chamber maintained at 24 $^{\circ }$C, with 45% relative humidity and an airflow of 0.4 m s$^{-1}$. Lighting was provided by full-spectrum adjustable LED lights (4000 W) on a 16-h light/8-h dark cycle.

Seedling establishment was evaluated 21 days after 50% of seedling emergence in the control groups^37^. Establishment rates and plant height were recorded. Throughout the study, differences in chlorophyll levels were assessed visually as leaf yellowing^51,52^. At 120 days the flowering status and health of the plants were assessed. Plants were harvested and excised below the second node, tissue from 2nd node to highest leaf was placed in an envelope, dried at 55$^\circ$C overnight, and weighed to determine above-ground dry biomass. Seed yield was quantified by counting all mature seeds per plant and weighing them after drying at $55^{\circ }\hbox {C}$ overnight. The standardized 100 seed weight was calculated by dividing the total dry seed weight (g) by the number of seeds produced and multiplying by 100. Mature plants were excised below the root radicle, and assessed for mycorrhization. Mycorrhization was confirmed using a modified trypan blue staining protocol^53^. Root samples were cleared in 5% potassium hydroxide (KOH) at 90$^\circ$C for 10 min, acidified in 1% hydrochloric acid (HCl) for 10 minutes, and stained with 0.10% trypan blue in lactoglycerol. Stained roots were examined under a compound microscope, where structures such as arbuscules, vesicles, and hyphae confirmed successful colonization. Substrate pH was measured at time zero, and after harvest with an Accumet Basic AB15 pH meter (Fisher Scientific, Hampton, NH, USA) calibrated with standard buffers (pH 4.0, 7.0, and 10.0).

After harvest, substrate aggregation was measured using the SLAKES app^43,54^. The SLAKES app captures the slaking process and uses image recognition to measure the projected aggregate area change over time. The SLAKES test provides a stability value ranging from 0.1 to 1. Higher values indicate greater aggregate stability and ability to maintain structure under stress, while values close to 0.1 suggest weaker structural integrity, with aggregates more likely to fall apart. We tested three aggregates per mixture, with tests repeated eight times.

All analyses were performed in MATLAB (MathWorks, Natick, MA). One–way ANOVA was used to test for treatment effects, and Tukey’s HSD for post hoc pairwise comparisons. Groups not significantly different ($p<0.05$) were indicated with shared letters in the figures.

## Supplementary Information

Supplementary Information.

## Data Availability

All data accompanying this manuscript can be found in Atkin, J., & Oliveira Pedro dos Santos, S. (2025). Bioremediation of Lunar Regolith Simulant Through Mycorrhizal Fungi and Plant Symbioses Enables Chickpea to Seed [Data set]. Zenodo. https://doi.org/10.5281/zenodo.17252917

## References

[1] Heiken, G., Vaniman, D. & French, B.M. Lunar Sourcebook: A User’s Guide to the Moon (1259) (1991).

[2] Ferl, R. J. & Paul, A.-L. Lunar plant biology-A review of the Apollo era. *Astrobiology* **10**(3), 261–274 (2010). 20446867 10.1089/ast.2009.0417

[3] Johnson, C. M. et al. Supplemental food production with plants: A review of NASA research. *Front. Astron. Sp. Sci.* **8**, 734343 (2021).

[4] Atkin, J. Using fungi for the bioremediation of lunar regolith. *Nat. Rev. Earth Environ.* 1–1 (2025).

[5] Duri, L.G., Romano, I., Adamo, P., Rouphael, Y., Pannico, A., Ventorino, V., Pepe, O., De Pascale, S. & Caporale, A.G. From earth to space: How bacterial consortia and green compost improve lettuce growth on lunar and Martian simulants. *Biol. Fertil. Soils* 1–20 (2025).

[6] Duri, L. G. et al. The potential for lunar and Martian regolith simulants to sustain plant growth: A multidisciplinary overview. *Front. Astron. Sp. Sci.* **8**, 747821 (2022).

[7] Ming, D. & Henninger, D. Use of lunar regolith as a substrate for plant growth. *Adv. Sp. Res.* **14**(11), 435–443 (1994). 10.1016/0273-1177(94)90333-6 11538023

[8] Walkinshaw, C. H., Sweet, H., Venketeswaran, S. & Horne, W. Results of Apollo 11 and 12 quarantine studies on plants. *BioScience* **20**(24), 1297–1302 (1970).

[9] Baur, P. et al. Uptake and translocation of elements from Apollo 11 lunar material by lettuce seedlings (1974).

[10] Walkinshaw, C. H. & Johnson, P. H. Analysis of vegetable seedlings grown in contact with Apollo 14 lunar surface fines. *HortScience* **6**(6), 532–535 (1971).

[11] Paul, A.-L., Elardo, S. M. & Ferl, R. Plants grown in Apollo lunar regolith present stress-associated transcriptomes that inform prospects for lunar exploration. *Commun. Biol.* **5**(1), 382 (2022). 35552509 10.1038/s42003-022-03334-8 PMC9098553

[12] Tabuchi, Y., Kioka, A. & Yamada, Y. Water permeability of sunlit lunar highlands regolith using LHS-1 simulant. *Acta Astron.* **213**, 344–354 (2023).

[13] Gilrain, M. R., Hogan, J. A., Cowan, R. M., Finstein, M. S. & Logendra, L. S. Preliminary study of greenhouse grown swiss chard in mixtures of compost and mars regolith simulant. In Technical Report, SAE Technical Paper (1999).

[14] Caporale, A. G. et al. Geo-mineralogical characterisation of mars simulant mms-1 and appraisal of substrate physico-chemical properties and crop performance obtained with variable green compost amendment rates. *Sci. Tot. Environ.* **720**, 137543 (2020). 10.1016/j.scitotenv.2020.137543 32135285

[15] Wamelink, G., Frissel, J., Krijnen, W. & Verwoert, M. Crop growth and viability of seeds on mars and moon soil simulants. *Terraform. Mars* 313–329 (2021).

[16] Roane, T. M., Pepper, I. L. & Gentry, T. J. Microorganisms and metal pollutants. In *Environ. Microbiol.* 415–439 (Elsevier, 2015).

[17] More, T., Yadav, J. S. S., Yan, S., Tyagi, R. D. & Surampalli, R. Y. Extracellular polymeric substances of bacteria and their potential environmental applications. *J. Environ. Manag.* **144**, 1–25 (2014). 10.1016/j.jenvman.2014.05.010 24907407

[18] Li, W.-W. & Yu, H.-Q. Insight into the roles of microbial extracellular polymer substances in metal biosorption. *Bioresour. Technol.* **160**, 15–23 (2014). 24345430 10.1016/j.biortech.2013.11.074

[19] Singh, G., Pankaj, U., Chand, S. & Verma, R. K. Arbuscular mycorrhizal fungi-assisted phytoextraction of toxic metals by Zea mays L. from tannery sludge. *Soil Sedim. Contamin. Int. J.* **28**(8), 729–746 (2019).

[20] Joner, E. J., Briones, R. & Leyval, C. Metal-binding capacity of arbuscular mycorrhizal mycelium. *Plant Soil* **226**, 227–234 (2000).

[21] Moland, S., Robicheau, B. M., Browne, R., Newell, R. & Walker, A. K. Determining the effects of biochar and an arbuscular mycorrhizal inoculant on the growth of fowl mannagrass (Glyceria striata) (Poaceae). *Facets* **3**(1), 441–454 (2018).

[22] Gujre, N., Soni, A., Rangan, L., Tsang, D. C. & Mitra, S. Sustainable improvement of soil health utilizing biochar and arbuscular mycorrhizal fungi: A review. *Environ. Pollut.* **268**, 115549 (2021). 33246313 10.1016/j.envpol.2020.115549

[23] Tisdall, J. M. & OADES, J. M. Organic matter and water-stable aggregates in soils. *J. Soil Sci.* **33**(2), 141–163 (1982).

[24] Miller, R. & Jastrow, J. Mycorrhizal fungi influence soil structure. In *Arbuscular Mycorrhizas: Physiology and Function*. 3–18 (2000).

[25] Dexter, A. Compression of soil around roots. *Plant Soil* **97**, 401–406 (1987).

[26] Dorioz, J. M., Robert, M. & Chenu, C. The role of roots, fungi and bacteria on clay particle organization. An experimental approach. In *Soil Structure/Soil Biota Interrelationships* (eds Beare, M. H. et al.) 179–194 (Elsevier, 1993).

[27] Douds, D. D. Jr., Pfeffer, P. E. & Shachar-Hill, Y. Carbon partitioning, cost, and metabolism of arbuscular mycorrhizas. In *Arbuscular Mycorrhizas: Physiology and Function* (eds Kapulnik, Y. & Douds, D. D.) 107–129 (Springer, 2000).

[28] Rillig, M. C., Wright, S. F. & Eviner, V. T. The role of arbuscular mycorrhizal fungi and glomalin in soil aggregation: Comparing effects of five plant species. *Plant Soil* **238**, 325–333 (2002).

[29] Cho, E. J. et al. Effects of AMF inoculation on growth of Panax ginseng CA Meyer seedlings and on soil structures in mycorrhizosphere. *Sci. Horticult.* **122**(4), 633–637 (2009).

[30] Sinha, R. K. et al. The wonders of earthworms & its vermicompost in farm production: Charles Darwin’s ‘friends of farmers’, with potential to replace destructive chemical fertilizers. *Agricult. Sci.* **1**(02), 76 (2010).

[31] Broyan Jr, J.L., Chu, A. & Ewert, M.K. Logistics reduction and repurposing technology for long duration space missions. In *International Conference on Environmental Systems* (2014).

[32] Hoff, J., Howe, J. & Mitchell, C.A. Nutritional and cultural aspects of plant species selection for a controlled ecological life support system. In *Technical Report* (1982).

[33] Atkin, J. et al. Genotype selection and microbial partnerships influence chickpea establishment in lunar regolith simulant. *Front. Astron. Sp. Sci.* **12**, 1670807.

[34] Islam, M. R. et al. Physiological responses of chickpea (Cicer arietinum) against chromium toxicity. *Rhizosphere* **24**, 100600 (2022).

[35] Velez, P. A., Talano, M. A., Paisio, C. E., Agostini, E. & González, P. S. Synergistic effect of chickpea plants and mesorhizobium as a natural system for chromium phytoremediation. *Environ. Technol.* **38**(17), 2164–2172 (2017). 27788623 10.1080/09593330.2016.1247198

[36] Naz, H. et al. Mesorhizobium improves chickpea growth under chromium stress and alleviates chromium contamination of soil. *J. Environ. Manag.* **338**, 117779 (2023). 10.1016/j.jenvman.2023.117779 37023603

[37] Wilhelm, K.-P. & Maibach, H. I. Oecd guidelines for testing of chemicals. In *Dermatotoxicology* 509–511 (CRC Press, 2012).

[38] Zaets, I. et al. Bioaugmentation in growing plants for lunar bases. *Adv. Sp. Res.* **47**(6), 1071–1078 (2011).

[39] Deepagoda, T. C. et al. Modeling gravity effects on water retention and gas transport characteristics in plant growth substrates. *Adv. Sp. Res.* **54**(4), 797–808 (2014).

[40] Liang, G. Iron uptake, signaling, and sensing in plants. *Plant Commun.* **3**(5) (2022). 10.1016/j.xplc.2022.100349 PMC9483112 35706354

[41] Marschner, H. *Marschner’s Mineral Nutrition of Higher Plants* (Academic Press, 2011).

[42] Kochian, L. V., Piñeros, M. A., Liu, J. & Magalhaes, J. V. Plant adaptation to acid soils: The molecular basis for crop aluminum resistance. *Annu. Rev. Plant Biol.* **66**, 571–598 (2015). 25621514 10.1146/annurev-arplant-043014-114822

[43] Fajardo, M., McBratney, A. B., Field, D. J. & Minasny, B. Soil slaking assessment using image recognition. *Soil Tillage Res.* **163**, 119–129 (2016).

[44] Fajardo, M. et al. Measuring soil aggregate stability with mobile phones, lessons, challenges, and future work. *Sci. Rep.* **15**(1), 24536 (2025). 40628934 10.1038/s41598-025-09925-9 PMC12238449

[45] Taylor, L. A., Pieters, C. M. & Britt, D. Evaluations of lunar regolith simulants. *Planet. Sp. Sci.* **126**, 1–7 (2016).

[46] Cannon, K. & Britt, D. Mineralogically accurate simulants for lunar ISRU, and strategic regolith processing. In *Lunar ISRU 2019-Developing a New Space Economy Through Lunar Resources and Their Utilization*. Vol. 2152. 5002 (2019).

[47] Long-Fox, J. M., Landsman, Z. A., Easter, P. B., Millwater, C. A. & Britt, D. T. Geomechanical properties of lunar regolith simulants LHS-1 and LMS-1. *Adv. Sp. Res.* **71**(12), 5400–5412 (2023).

[48] Lucas, M., Neal, C., Long-Fox, J. & Britt, D. Replicating the geotechnical properties of lunar highland regolith stratigraphy using high-fidelity LHS-1 simulant. *LPI Contrib.* **2678**, 2687 (2022).

[49] Isachenkov, M. et al. Characterization of novel lunar highland and mare simulants for ISRU research applications. *Icarus* **376**, 114873 (2022).

[50] Farooq, M., Ullah, A., Lee, D.-J., Alghamdi, S. S. & Siddique, K. H. Desi chickpea genotypes tolerate drought stress better than Kabuli types by modulating germination metabolism, trehalose accumulation, and carbon assimilation. *Plant Physiol. Biochem.* **126**, 47–54 (2018). 29499435 10.1016/j.plaphy.2018.02.020

[51] Yadav, S. P., Ibaraki, Y. & Dutta Gupta, S. Estimation of the chlorophyll content of micropropagated potato plants using rgb based image analysis. *Plant Cell Tissue Organ Cult. (PCTOC)* **100**, 183–188 (2010).

[52] Ougham, H. et al. The control of chlorophyll catabolism and the status of yellowing as a biomarker of leaf senescence. *Plant Biol.* **10**, 4–14 (2008). 18721307 10.1111/j.1438-8677.2008.00081.x

[53] Giovannetti, M. & Mosse, B. An evaluation of techniques for measuring vesicular arbuscular mycorrhizal infection in roots. *New Phytol.* 489–500 (1980).

[54] Flynn, K. D., Bagnall, D. K. & Morgan, C. L. Evaluation of slakes, a smartphone application for quantifying aggregate stability, in high-clay soils. *Soil Sci. Soc. Am. J.* **84**(2), 345–353 (2020).

