# Supplementary material for: Bioremediation of lunar regolith simulant through mycorrhizal fungi and plant symbioses enables chickpea to seed
Source: Sci Rep. 2026 Mar 5;16:7498. doi: 10.1038/s41598-026-35759-0 (PMC12963618; doi:10.1038/s41598-026-35759-0)
Supplement: Supplementary file 1 — Supplementary Information. [file 41598_2026_35759_MOESM1_ESM.pdf]

Supplementary Information

Bioremediation of Lunar Regolith Simulant through Mycorrhizal Fungi and Plant Symbioses Enables Chickpea to Seed

Jessica Atkin, Elizabeth Pierson, Terry Gentry, Sara Oliveira Santos

Supplementary Figures

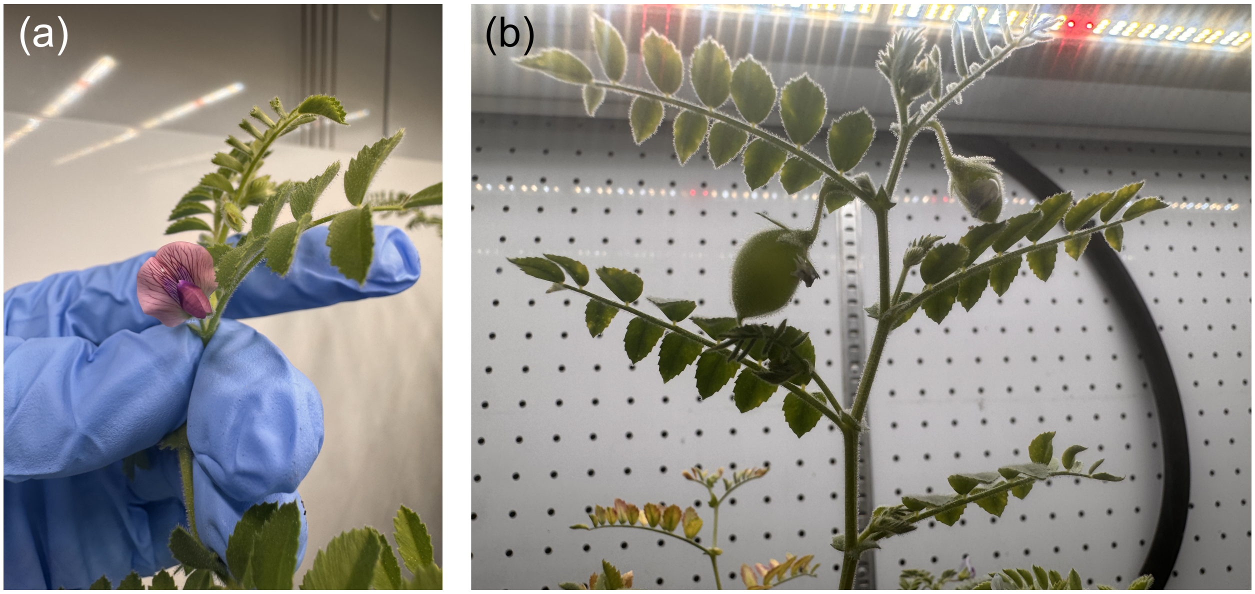

Figure 1: ***Flowering and seed set in LRS75***. *(a) Flowering in LRS75. (b) Seed set in LRS75.*

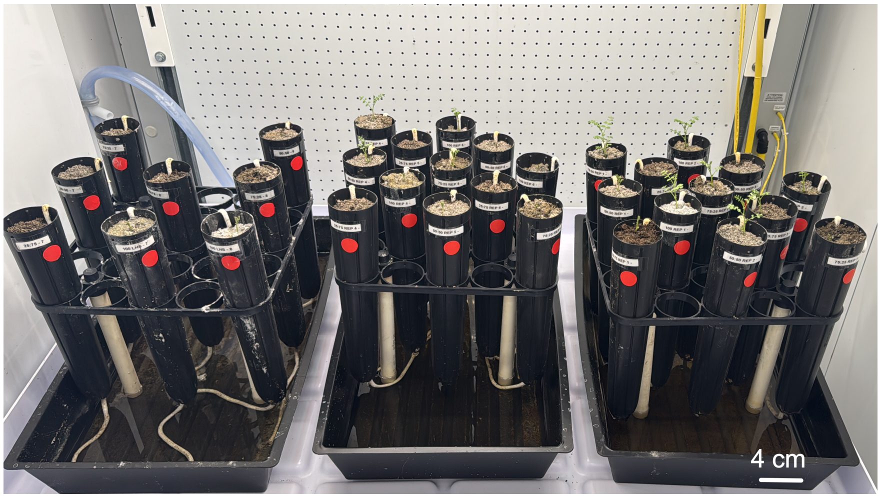

Figure 2: ***Randomized block design.*** *Chickpeas were planted in LRS mixtures in 35 cm tall containers. Watering was done through cotton wicks dipped in water trays. Scale bar is 4 cm.*

Table 1: *Physical, mineralogical, and geotechnical properties of the LHS-1 Highlands Simulant. Note: Values measured by XRF; totals may not equal 100 wt % due to rounding. Data adapted from sources listed below.*[1]

**Category**	**Property**	**Value**
General	Simulant Name	LHS-1 Highlands Simulant
	Simulant Type	General purpose
	Reference Material	Average lunar highlands
Physical	Uncompressed Bulk Density	1.27 $g\,cm^{-3}$
	Median Particle Size	81.62 μm
	Particle Size Range	<0.04 μm – 1000 μm
Mineralogy	Anorthosite	74.4 wt%
	Basalt	24.7 wt%
	Ilmenite	0.4 wt%
	Bronzite	0.3 wt%
	Olivine	0.2 wt%
	SiO_2	49.12 wt%
	TiO_2	0.63 wt%
	Al_2O_3	26.29 wt%
	FeO	3.20 wt%
	MnO	0.06 wt%
	MgO	2.86 wt%
	CaO	13.52 wt%
	Na_2O	2.55 wt%
	K_2O	0.34 wt%
	P_2O_5	0.17 wt%
	LOI	0.41 wt%
	Total	99.15 wt%
Geotechnical	Angle of Repose	39.89°
	Angle of Repose	38.45°
	Cohesion	0.311 kPa
	Angle of Internal Friction	31.49°

[1] Adapted from [1, 2, 3].

References

[1] Lawrence A Taylor, Carle M Pieters, and Daniel Britt. Evaluations of lunar regolith simulants. *Planetary and Space Science*, 126:1–7, 2016.

[2] KM Cannon and DT Britt. Mineralogically accurate simulants for lunar isru, and strategic regolith processing. *Lunar ISRU 2019-Developing a New Space Economy Through Lunar Resources and Their Utilization*, 2152:5002, 2019.

[3] Jared M Long-Fox, Zoe A Landsman, Parks B Easter, Catherine A Millwater, and Daniel T Britt. Geomechanical properties of lunar regolith simulants lhs-1 and lms-1. *Advances in Space Research*, 71(12):5400–5412, 2023.
